# Genetic Correlation, Genome-Wide Association and Genomic Prediction of Portable NIRS Predicted Carotenoids in Cassava Roots

**DOI:** 10.3389/fpls.2019.01570

**Published:** 2019-12-04

**Authors:** Ugochukwu N. Ikeogu, Deniz Akdemir, Marnin D. Wolfe, Uche G. Okeke, Amaefula Chinedozi, Jean-Luc Jannink, Chiedozie N. Egesi

**Affiliations:** ^1^Plant Breeding and Genetics Section, Cornell University, Ithaca, NY, United States; ^2^Biotechnology Department, National Root Crops Research Institute, Umudike, Nigeria; ^3^Cornell University Statistical Consulting Unit (CSCU), Cornell University, Ithaca, NY, United States; ^4^Plant, Soil and Nutrition Research, Robert W. Holley Center for Agriculture & Health, Agricultural Research Service, United States Department of Agriculture (USDA), Ithaca, NY, United States; ^5^Cassava Breeding Department, International Institute of Tropical Agriculture (IITA), Ibadan, Nigeria

**Keywords:** cassava, carotenoids, genome-wide association studies (GWAS), genomic selection (GS), calibration, near infra-red spectroscopy (NIRS)

## Abstract

Random forests (RF) was used to correlate spectral responses to known wet chemistry carotenoid concentrations including total carotenoid content (TCC), all-trans β-carotene (ATBC), violaxanthin (VIO), lutein (LUT), 15-cis beta-carotene (15CBC), 13-cis beta-carotene (13CBC), alpha-carotene (AC), 9-cis beta-carotene (9CBC), and phytoene (PHY) from laboratory analysis of 173 cassava root samples in Columbia. The cross-validated correlations between the actual and estimated carotenoid values using RF ranged from 0.62 in PHY to 0.97 in ATBC. The developed models were used to evaluate the carotenoids of 594 cassava clones with spectral information collected across three locations in a national breeding program (NRCRI, Umudike), Nigeria. Both populations contained cassava clones characterized as white and yellow. The NRCRI evaluated phenotypes were used to assess the genetic correlations, conduct genome-wide association studies (GWAS), and genomic predictions. Estimates of genetic correlation showed various levels of the relationship among the carotenoids. The associations between TCC and the individual carotenoids were all significant (P < 0.001) with high positive values (r > 0.75, except in LUT and PHY where r < 0.3). The GWAS revealed significant genomic regions on chromosomes 1, 2, 4, 13, 14, and 15 associated with variation in at least one of the carotenoids. One of the identified candidate genes, phytoene synthase (PSY) has been widely reported for variation in TCC in cassava. On average, genomic prediction accuracies from the single-trait genomic best linear unbiased prediction (GBLUP) and RF as well as from a multiple-trait GBLUP model ranged from ∼0.2 in LUT and PHY to 0.52 in TCC. The multiple-trait GBLUP model gave slightly higher accuracies than the single trait GBLUP and RF models. This study is one of the initial attempts in understanding the genetic basis of individual carotenoids and demonstrates the usefulness of NIRS in cassava improvement.

## Introduction

Carotenoids are well known for their nutritional and health benefits, particularly in the prevention of a number of human cancers and eye diseases. Most important is the vitamin A activity of the provitamin A carotenoids (PVAC), especially, beta-carotene, alpha-carotene, beta-cryptoxanthin, and gamma-carotene (Krinsky and Johnson, 2005; Paiva and Russell, 2013). Vitamin A is essential for growth and differentiation of a number of cells and tissues and vital for the healthy development of the fetus and the newborn (Strobel et al., 2007). Inadequate intake of vitamin A is associated with impaired vision, poor immunity, retarded growth, and even death, particularly among children and pregnant or nursing mothers (Strobel et al., 2007; Ceballos et al., 2013; Bechoff et al., 2015). Also, carotenoids act as antioxidants and some non-PVAC, for example, lutein and zeaxanthin, are important components of the macular pigment in the eyes, and their deficiencies are linked to some eye-related problems (Krinsky and Johnson, 2005; Kim et al., 2010; Bechoff et al., 2015).

Cassava is the fourth most important basic food after rice, wheat, and maize worldwide and provides food for many people, particularly in sub-Saharan Africa, where over 600 million people depend on it to meet their energy requirements (Oliveira et al., 2014; Rabbi et al., 2017). Generally, cassava roots are low in nutritional quality, containing mainly carbohydrates (Nweke, 2004; Ceballos et al., 2017). However, there are ongoing efforts to improve the nutritional quality of cassava, taking advantage of genetic variability existing in the crop (Ceballos et al., 2013; Mugode et al., 2014; Ceballos et al., 2017). Such efforts are invaluable in alleviating vitamin A deficiency (VAD) problems prevalent among individuals below poverty thresholds who cannot afford healthy and balanced nutrition from more expensive food sources (Maziya-Dixon et al., 2006; Strobel et al., 2007). The bio-fortification effort has led to a substantial boost in the proportion of carotenoids in cassava roots and the recorded success has been largely attributed to the adoption of advanced analytical tools (Marini et al., 2013; Belalcazar et al., 2016).

In cassava phenotyping, the conventional use of color intensity in quantifying carotenoid content in cassava roots is challenging and restricted to a qualitative classification of clones into white, cream and yellow categories (Ceballos et al., 2017). Other alternatives include the use of high-performance liquid chromatography (HPLC) and UV-Visible spectrophotometry, which are low-throughput and require skilled labor and favorable laboratory conditions (Ceballos et al., 2013; Belalcazar et al., 2016). Such laboratory facilities and conditions are lacking in low-resource breeding programs and experimental sites (out-stations) where most multilocation evaluations take place. Besides, such standard facilities are expensive to install and ineffective for large volume analytical procedures. Recently, the use of near infra-red spectroscopy (NIRS) has been demonstrated to enable high-throughput assessment and quantitative evaluation of micro-nutrients including total and individual carotenoid components (Sánchez et al., 2014; Belalcazar, et al., 2016; Ikeogu et al., 2017). Such development is necessary for accurate phenotyping and understanding of the underlying genetics of PVAC in cassava.

Linear regression models have been widely used in developing NIRS calibrations—correlating spectral response of each sample at individual wavelengths to known chemical concentrations from laboratory analysis (Chen and Wang, 2001), but their performance is often limited by nonlinear effects including baseline drift, light scattering effects, and multicollinearity (Büning-Pfaue and Kehraus, 2001; Cen and He, 2007; Sánchez et al., 2014). Linear models generally perform regression on factor analysis components which in many cases, lack direct physical meaning (Cristianini and Shawe-Taylor, 2000; Wold et al., 2001; Andersson, 2009; Ghasemi and Tavakoli, 2013). Recently, the option of nonlinear calibration models has been gaining attention as such models are useful in addressing both linear and nonlinear multivariate relationships. The growing interest in the use of nonlinear models for spectra analyses could be attributed to their comparable accuracy, mathematical simplicity, computational efficiency, and robustness to noise (Breiman, 2001; Lee et al., 2012; Ghasemi and Tavakoli, 2013). Random forests (RF), a nonlinear model, has been effective in multivariate calibrations from modern measuring instruments, including spectrometers, chromatographs, and sensor batteries where it has been used to provide valuable interpretable results. It also provides an adequate fine-tuning mechanism to control overfitting and collinearity associated with most spectroscopic data (Svetnik et al., 2003; Ghasemi and Tavakoli, 2013; Sila et al., 2016).

The lack of adequate phenotyping tools especially in dissecting total carotenoid content (TCC) into its individual components is a limiting factor in the genetic studies of PVAC in cassava. Genome-wide association studies (GWAS), which leverage available marker polymorphisms distributed throughout the cassava genome, have been useful in identifying the genomic regions associated mainly with TCC variation in cassava (Esuma et al., 2016; Rabbi et al., 2017). GWAS could fill in the limited information on the genomic regions associated with most of the individual carotenoids, their relative genetic control, and correlations.

Naturally, carotenoids are present in various configurations and isomerization (Castenmiller and West, 1998; Paiva and Russell, 2013). In addressing VAD, attention should be given to the reported bioavailability and bioconversion interactions of carotenoid components including a positive interaction between β-carotene and concentrations of α-carotene, negative interactions between β-carotene and lutein, lycopene, and canthaxanthin (Castenmiller and West, 1998). From a breeding perspective, it is very important to establish the genetic correlations of carotenoid components in cassava and determine the relationship between such correlations and the reported bioavailability and bioconversion interactions (Castenmiller and West, 1998; Strobel et al., 2007; Mugode et al., 2014; Bechoff et al., 2015). In addition, understanding the relationships between TCC and the individual components will help to track the extent of progress made thus far or need to be made, including the adoption of the best strategy for carotenoids improvement in cassava roots.

Unlike GWAS, genomic selection (GS) is a breeding technology that is used to predict the genetic potential of individuals in a breeding program without necessarily uncovering the underlying genes and quantitative trait loci (QTL) behind the traits of interest (Meuwissen et al., 2001; Goddard and Hayes, 2007; VanRaden, 2008). It promises to accelerate genetic gain over time, shorten breeding cycles and reduce the costs of breeding (Habier et al., 2009; Hayes et al., 2010; de Oliveira et al., 2012; Wolfe et al., 2017). As the field continues to grow and new computational methods develop, nonlinear GS models have been shown to be useful in estimating total genetic values (TGVs) beyond just breeding values (Lorenz et al., 2011; Heslot et al., 2012; Wolfe et al., 2017). Being a clonally propagated crop, the TGV of a cassava plant can be reproduced so that its prediction from nonlinear GS prediction models is appropriate in cassava breeding and trait improvement.

Laboratory facilities to assay the full suite of carotenoids are not readily available in Nigeria. In order to assess the full spectrum of carotenoids in Nigerian cassava germplasm, we leveraged a calibration population developed at the International Center for Tropical Agriculture (CIAT) in Cali-Palmira, Colombia, to predict content of carotenoids in a cassava population of the National Root Crops Research Institute (NRCRI) in Umudike, Nigeria. We validated the predictions by assessing their genotype to phenotype relationships in terms of heritability, genomic prediction accuracy, and the identification of significant GWAS hits. We used RF, a nonlinear method for NIRs prediction for TCC, ATBC, VIO, LUT, 15CBC, 13CBC, AC, 9CBC, and PHY in cassava and employed the calibration models in analyzing the spectral information of a training population from NRCRI in Nigeria. We estimated the genetic correlations, identified the underlying genomic regions associated with the variation, and demonstrated the potential of using GS for the rapid improvement of these traits, comparing linear with nonlinear prediction models. While many GS predictions are performed on a single trait basis, the use of multiple-trait models has shown prediction improvements in various cases (Jia and Jannink, 2012; Fernandes et al., 2017; Okeke et al., 2017). Therefore, we also compared predictions of single and multiple-trait GS models for the improvement of carotenoids in fresh cassava roots.

## Materials and Methods

### Training Population and Spectra Collection

NRCRI has a training population currently used for the implementation of GS in cassava which has been fully described (Wolfe et al., 2016; Wolfe et al., 2017). The germplasm consists of Training Population 1 (TP1) and Training Population 2 (TP2). Trials of these two populations were further divided into sets (TP1 had two sets and TP2 had four sets) for easy management and the control of experimental error and the sets in each trial were established as randomized incomplete blocks with three replications of a plot size of five plants. TP1 was evaluated at Umudike in a single set whereas TP2 was evaluated at Umudike, Otobi, and Kano using four sets in the 2015/2016 cropping season. Two or three technical replications were taken in each clone replication across sets and trials. A total of 594 clones from the two populations—221 (TP1) and 411 (TP2) with an overlap of 24 clones, were used for analyses. The origin of the NRCRI clones has been described (Wolfe et al., 2016). Briefly, most of the clones have ancestry from germplasm introduced from the International Center for Tropical Agriculture (CIAT), Cali-Palmira, Colombia (Njoku et al., 2011; Ceballos et al., 2013). Also, a cluster analysis of spectral data from CIAT and NRCRI roots (data not shown) did not suggest the two populations were disjoint. The training population included clones characterized as white and few others as yellow provitamin A clones.

Spectral data on the TP were collected using a full range (350 – 2500 nm wavelength in 1-nm increments) portable visible and infrared spectrometer (Vis/NIRS) (QualitySpec Trek: S-10016, ASD Inc.). Root samples from two to three sizeable roots were randomly selected from a plot and the selected roots were peeled with knives, washed, and homogenized into a paste-like mash using a portable power-operated grater. Spectral data were collected from homogenized mashed samples in quartz sampling cups placed against the window of the portable Vis/NIRS device. Each final spectral output was a mean of fifty scans (Ikeogu et al., 2017).

### Training Population Carotenoids Phenotype Evaluation

The carotenoids of NRCRI training population were estimated from calibration equations derived from a calibration population (n = 173) developed from the breeding population of CIAT using RF. Usually, the use of NIR instruments for analyses require the training, also known as the calibration of the instruments for the evaluation of traits of interest. Calibration establishes a mathematical relationship between the absorption spectra from the NIR instruments and the factor of interest (Chen and Wang, 2001; Cen and He, 2007). Developing a calibration model requires spectra measurements of samples from a population that includes all variances in future prediction and some important aspects of calibration development require using a good number of samples uniformly covering a wide range of the analytes of interest from the calibration set known as a training set to develop models. Thereafter, the developed calibration models should be validated to test the model performance on future samples on the remaining subset of the calibration set (test set) (Cen and He, 2007; Lopez et al., 2013). The calibration population has been previously described and analyzed using a linear calibration model—modified partial least square regression, with mashed cassava root samples and HPLC reference values (Ikeogu et al., 2017). Just like the NRCRI population, the calibration population contained clones characterized as both white and yellow. Calibration was performed in R using the *caret* package (Kuhn, 2008; R Core Team, 2017).

Prior to building calibration models, standard normal variate and detrending (SNVD) spectra pretreatment (D = 2, G = 5, S1 = 2, S2 = 1) was applied to correct for external interferences on the spectral data, where D indicates the derivative order number (0 indicates no derivation, 1 means the first derivative, and so on), G indicates the gap (the number of data points over which derivation is computed), S1 indicates the number of data points in the first smoothing (1 means no smoothing), and S2 indicates the number of data points in the second smoothing (1 means no smoothing) (Davrieux et al., 2016; Ikeogu et al., 2017).

The cross-validation of the NIRS calibration models was done by dividing the calibration set into training and testing sets in a ratio of 3:1 which was repeated 10 times. After assessing the performance of the initial calibration models on the testing set, a final model was fitted on the full calibration set for each trait in order to maximize the number of calibration samples. These final RF models were used in predicting the carotenoids of over 4,000 spectra from 594 clones from NRCRI across Umudike, Otobi, and Kano locations.

### Genotype Data

The genotype data used in this study have been previously described (Wolfe et al., 2016; Wolfe et al., 2017). The data were generated using genotyping by sequencing (GBS) with the ApeK1 restriction enzyme. SNP calls were carried out with the TASSEL GBS pipeline V4 (Glaubitz et al., 2014) and aligned to the cassava reference genome (Goodstein et al., 2012; Bredeson et al., 2016). Individuals with more than 80% missing SNP calls and markers with more than 60% missing calls were removed. Missing data were imputed with Beagle (version 4.0) (Browning and Browning, 2008) and marker data were then converted to a dosage format. After filtering based on MAF > 0.01, a total of 114,884 SNP markers were used for analyses.

### Trait Correlations and Deregressed BLUPS

The estimate of genetic correlations $\left(r_{G}^{2}\right)$ among the reported carotenoids was obtained by Pearson correlation of estimated genetic values (EGV) derived from a mixed linear model for each carotenoid response. The linear model was

y= μ+loc+clone+trial+set(loc:trial)+rep(set)+ε,

where y = the NIRS predicted phenotypes; µ = population mean; loc = fixed effect of location; clone = random effect of clone: $\text{clone}\sim \text{N(}0,\text{ I}\sigma_{\text{clone }}^{2})$; trial = fixed effect of trial; *set (loc:trial)* = random effect of set nested in trial and location: $set\sim N(0,\text{I}\sigma_{\text{set }}^{2}),$ rep(set) = random effect of clone replication nested in $set:rep\sim N(0,\text{I}\sigma_{rep}^{2})\;$ and ε = error term: $\varepsilon \sim N(0,\text{I}\sigma_{\varepsilon }^{2}).$ Models were fitted using the lmer package in R (Bates et al., 2014; R Core Team, 2017).

Since the clones were not replicated equally across locations, trials, and sets, our data set was unbalanced and in order to account for the variability in predicted error variance (PEV) and unequal shrinking of the BLUPS for clones, BLUPs were deregressed on the basis of PEV (Garrick et al., 2009). The deregressed BLUPs (dBLUPS) were used in the downstream studies. Broad-sense heritability (*H* ^2^) was calculated using the estimated variance components from the mixed models according to (Holland et al., 2010) as

$$H^{2}=\;\frac{\sigma_{C}^{2}}{\left(\sigma_{C}^{2}+\frac{\sigma_{S}^{2}}{\bar{s}}+\frac{\sigma_{r}^{2}}{\bar{r}}+\frac{\sigma_{e}^{2}\;}{\;\bar{rs}}\right)},$$

where, $\sigma_{C}^{2}$ = clone variance, $\sigma_{S}^{2}$=set variance, $\sigma_{r}^{2}$=replication variance, $\sigma_{e}^{2}$=error variance,while $\bar{s}$, $\bar{r}$ and $\bar{rs}$ were harmonic mean number of sets, mean number of replications, and mean number of plots in which each clone was observed, respectively.

### GS Models

#### Single Trait, ST-GBLUP, and ST-RF Models

Genomic estimated breeding values (GEBVs) for the clones were extracted using the genomic BLUP (GBLUP) model which is defined as

y=1μ+Zu+ε,

where $u\sim N(0,\sigma_{u\;}^{2}K),\varepsilon \sim N(0,I\sigma_{\varepsilon \;}^{2})$ and **y** is the vector of dBLUPs for each carotenoid; 1 is a vector of ones; *µ* is the mean for the dBLUP values; **u** is the vector of random additive genomic effects (GEBVs) with the corresponding design matrix Z; and K is the additive genomic relationship matrix calculated from SNPs using method 1 of (VanRaden, 2008). The ST-GBLUP models were fitted using the *sommer* package (Covarrubias-Pazaran, 2016). RF models were trained to estimate the TGVs. TGVs are different from GEBVs since they incorporate nonlinear genetic effects. The ST-RF model was carried out with the *randomForest* package in R (Breiman, 2001; Svetnik et al., 2003).

#### Multiple-Trait, MT-GBLUP Model

The nine carotenoids were modeled as multiple response in the multiple-trait model: *Y = M + ZU + E*, where *Y* is the response matrix of the dBLUPs for the nine carotenoids; M is the matrix for the means (*M=1µ'* where 1 is a vector of ones, and *µ* is the means vector for the nine carotenoids); U is a random matrix of additive genomic effects vector (GEBVs) with the design matrix Z and *E* is an independent residual matrix; U and E are assumed to have independent matrix variate normal distributions given as N(0, V_Z_, K) and N(0, V_ε_, I_n_) respectively. The multiple-trait GEBVs were derived using the *EMMREML* package in R (Akdemir and Okeke, 2015; R Core Team, 2017). Prediction accuracies were derived as the correlation between the deregressed EGV and the genetic value predicted by the marker-informed models using a fivefold cross-validation scheme (Kohavi, 1995) iterated 30 times.

#### Genome-Wide Association Analysis

A genome-wide association analysis to identify genetic variants associated with the NIRS predicted carotenoids was carried out using GCTA software (Yang et al., 2011). Markers were further filtered and 87,380 SNPs with MAF > 0.05 were retained for the analysis.

## Results

### Vis/NIRS Calibration and Carotenoids Analyses

The result of the initial calibration models with the 3:1, training:test sets showed that correlation between the actual and predicted values within the training set (rc) ranged from 0.66 in PHY to 0.97 in ATBC (**Table 1**). Similarly, the correlation between the actual values and predicted values in the test set (rcv), predicted using the model developed from the training set ranged from 0.62 in PHY to 0.97 in ATBC (**Table 1**). The root-mean-squared error (RMSE) was highest in PHY (2.9) and lowest in AC (0.01). In the final calibration models (combined set of training and test sets), the rc was similar to the initial calibration with only the training set (**Table 1**).

**Table 1:** Calibration statistics of the portable Vis/NIRS spectra analyzed using random forests for total carotenoid content (TCC), all-trans β-carotene (ATBC), violaxanthin (VIO), lutein (LUT), 15-cis beta-carotene (15CBC), 13-cis beta-carotene (13CBC), alpha-carotene (AC), 9-cis beta-carotene (9CBC), and phytoene (PHY) carotenoids in cassava roots.

Model	Stat.	TCC	AC	ATBC	LUT	VIO	9CBC	13CBC	15CBC	PHY
Cal.	r_c_	0.96	0.87	0.97	0.77	0.79	0.90	0.92	0.92	0.66
	r_cv_	0.96	0.86	0.97	0.73	0.77	0.89	0.91	0.91	0.62
	RMSE	2.65	0.01	1.6	0.32	0.14	0.26	0.38	0.06	2.9
	N_c_	132	59	132	84	132	132	132	131	71
Final	r_c_	0.95	0.85	0.96	0.75	0.76	0.88	0.89	0.91	0.52
	RMSE	2.51	0.01	1.6	0.33	0.14	0.26	0.33	0.06	2.8
	N_c_	173	76	173	109	173	173	173	173	91

r_c_ = correlation between predicted and actual values in training set; r_cv_ = correlation between predicted and actual values in test set; RMSE, root-mean-square error; N_c_ = number of observations in the training set.

### Statistical Summary and Heritability of Carotenoids From NRCRI Breeding Program

There was considerable phenotypic variation for all the carotenoids from the analyzed NRCRI TP data (**Table 2**). There was a number of both white and yellow root clones similar to a population earlier used for GWAS studies for TCC (Rabbi et al., 2017). The predicted TCC values ranged from 2 µgg^-1^ to 15.39 µgg^-1^ with an average of 4.72 µgg^-1^ (fresh weight basis). Variation in ATBC ranged from 0.53 µgg^-1^ to 10.18 µgg^-1^ with a mean of 1.58 µgg^-1^ (**Table 2**). Compared to other traits, AC had a narrower range of 0.05 µgg^-1^ to 0.07 µgg^-1^ with a mean of 0.06 µgg^-1^ and standard deviation of 0.004 (**Table 2**). The broad sense heritability for these traits ranged from 0.24 in LUT to 0.80 in TCC and 15CBC (**Table 2**).

**Table 2:** Summary statistics and heritability of total carotenoid content (TCC), all-trans β-carotene (ATBC), violaxanthin (VIO), lutein (LUT), 15-cis beta-carotene (15CBC), 13-cis beta-carotene (13CBC), alpha-carotene (AC), 9-cis beta-carotene (9CBC), and phytoene (PHY) from cassava roots.

Stat.	TCC	AC	ATBC	LUT	VIO	9CBC	13CBC	15CBC	PHY
Min.	2.20	0.05	0.53	0.14	0.22	0.23	0.28	0.05	3.68
Max.	15.39	0.07	10.18	1.45	0.61	1.15	1.44	0.26	8.99
Mean	4.72	0.06	1.58	0.25	0.33	0.44	0.56	0.10	5.41
SD	2.085	0.004	1.536	0.098	0.055	0.163	0.212	0.039	0.701
H^2^	0.8	0.65	0.81	0.24	0.61	0.79	0.78	0.8	0.71

### Genetic Correlation Among Carotenoids

**Figure 1:**
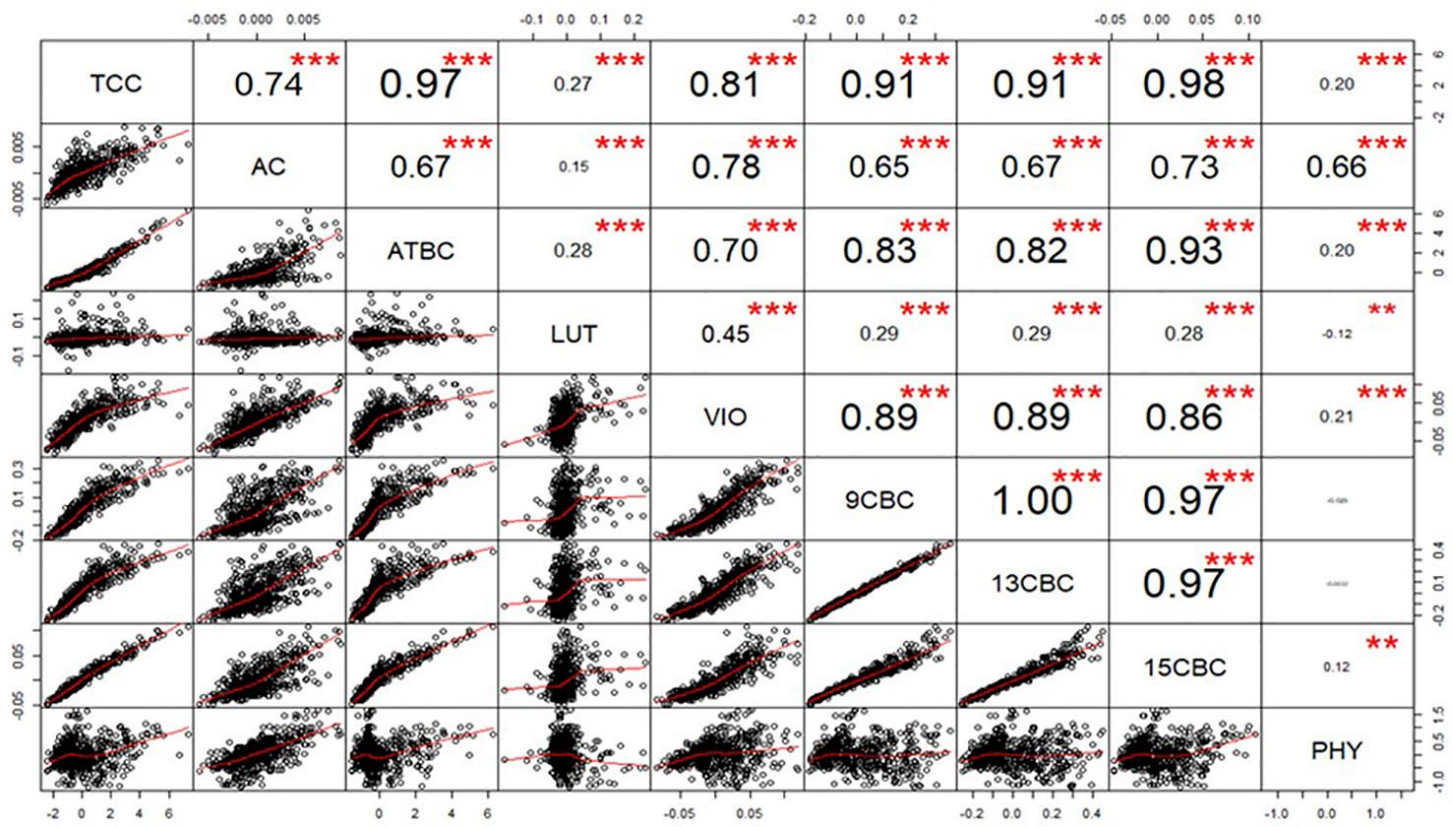
Genotypic correlation of total carotenoid content (TCC), all-trans β-carotene (ATBC), violaxanthin (VIO), lutein (LUT), 15-cis beta-carotene (15CBC), 13-cis beta-carotene (13CBC), alpha-carotene (AC), 9-cis beta-carotene (9CBC), and phytoene (PHY) carotenoids in cassava roots.

After calculating the genetic correlations, few values in LUT and VIO seem to be influencing the result (data not shown), we used a generalized extreme studentized deviate outlier test to identify and remove the extreme points and recalculated the correlations (**Figure 1**). The correlation between TCC and the individual components was highest in 15CBC (r = 0.98; p-value <0.001) and lowest in PHY (r = 0.18; p-value < 0.001) (**Figure 1**). Among the carotenoid components, the highest genetic correlation was observed between 9CBC and 13CBC (r ≈ 1). Other associations were mostly positive and highly significant (p < 0.001). However, a significant (p-value <0.001) and negative correlation was observed between PHY and LUT (r = -0.12). Negative but nonsignificant associations were recorded between PHY and 9CBC as well as 13CBC (**Figure 1**).

### Genome-Wide Association Studies

**Figure 2:**
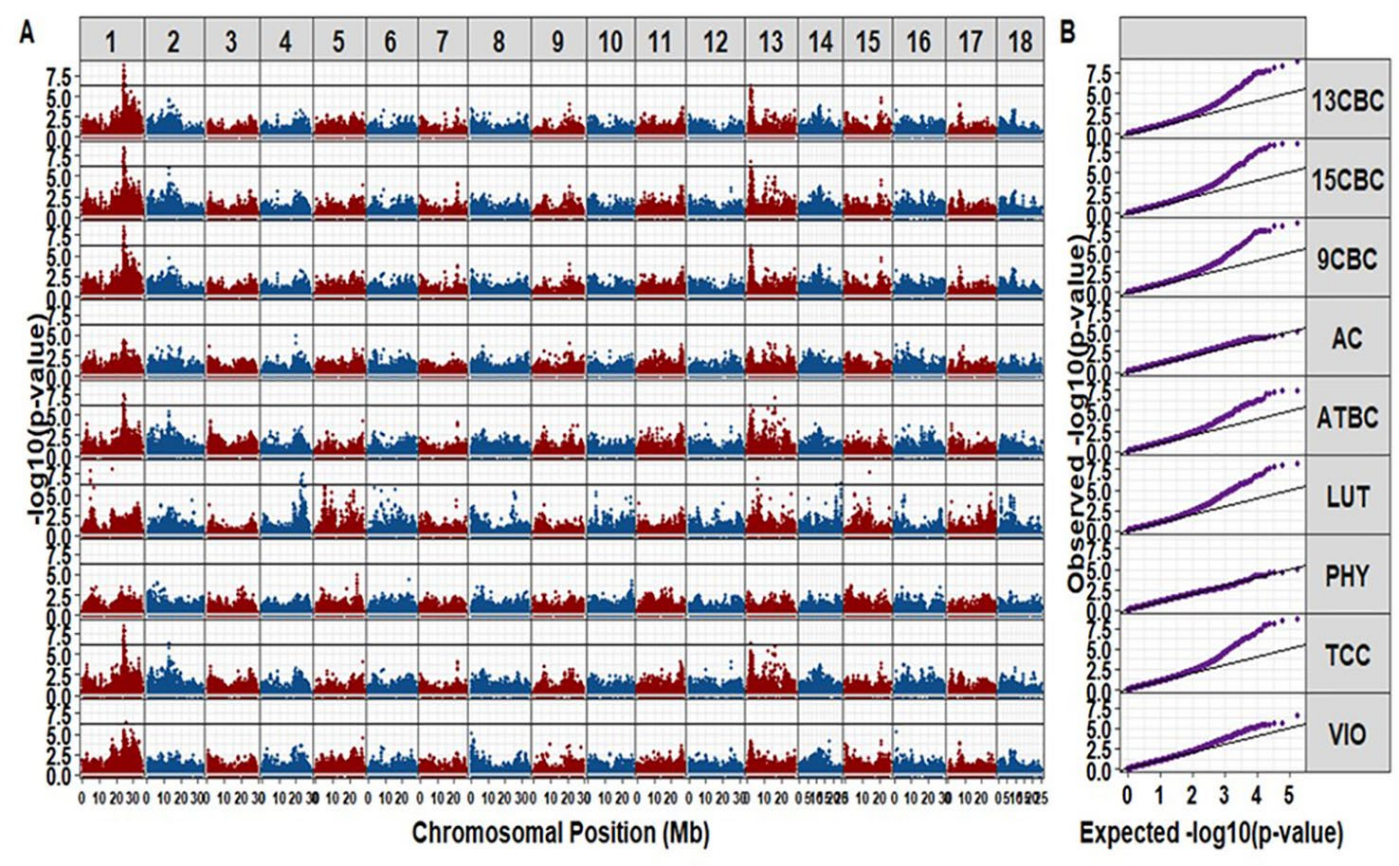
The Manhattan **(A)** and QQ **(B)** plots of genome-wide association studies on cassava root total carotenoid content (TCC), all-trans β-carotene (ATBC), violaxanthin (VIO), lutein (LUT), 15-cis beta-carotene (15CBC), 13-cis beta-carotene (13CBC), alpha-carotene (AC), 9-cis beta-carotene (9CBC), and phytoene (PHY) carotenoids.

We identified a total of 42 unique markers significantly associated with variation in TCC and individual carotenoids (i.e., with p-values small than a Bonferroni threshold at an alpha of 5%). Most of the significant markers were associated with variation in more than one trait (**Table S1**). There was no significant hit for AC and PHY from this study (**Figure 2**). The observed regions associated with variation in the different carotenoid components were on chromosomes 1, 2, 4, 13, 14, and 15. A total of 20 markers were significant for variation in TCC, and 17 of those markers were located between 23.386 Mbp to 24.709 Mbp on chromosome 1. A single marker tagged another peak around 12.739 Mbp on Chromosome 2 (p-value = 4.71 × 10^-7^) and the remaining two markers tagged another peak around 21.85 Mbp on chromosome 13 (p-value = 4.34 × 10^-7^) (**Table S1**). Interestingly, similar regions tagged by almost the same markers for variation in TCC were significant for variation in ATBC, 9CBC, 13CBC, and 15CBC. In addition, there was a nearby peak at 25.427 Mbp tagged by one marker (p-value = 3.50 × 10^-7^) and significant for variation in both 13CBC and VIO. On the other hand, five hits were associated with variation in LUT on chromosome 1, tagged by four significant markers of which three were localized between 4.81 Mbp and 4.86 Mbp and the remaining marker around 17.48 Mbp, five markers tagged a peak around 22.54 Mbp to 23.69 Mbp on chromosome 4 and a marker on each of chromosome 13 (6.09 Mbp and p-values = 1.04 x 10^-7^), chromosome 14 (24.24 Mbp and p-values = 3.28 x 10^-7^), and chromosome 15 (14.17 Mbp and p-values = 1.86 × 10^-8^).

The cassava genome (v6.1) (Bredeson et al., 2016) on Phytozome (v12.1.6) (Goodstein et al., 2012) was queried to identify annotated genes within 0.5 Mb of the genomic regions occupied by significant SNPs. The candidate gene *Manes.01G124200*, a phytoene synthase (PSY) gene known for increasing the accumulation of carotenoid in cassava roots (Welsch et al., 2010; Esuma et al., 2016; Rabbi et al., 2017) and *Manes.01G001200* gene also associated with carotenoid biosynthesis (Goodstein et al., 2012; Bredeson et al., 2016), located within the genomic regions (∼24.15 to 24.16 Mbp, forward, and 25.21 to 25.48 Mbp, forward, respectively) were found around the regions of the significant markers on chromosome 1, which was associated with variation in TCC, ATBC, 9CBC, 13CBC, 15CBC, and VIO. There were other noncarotenoid candidate genes (not reported) found in the other regions associated with variation in the studied carotenoids on chromosomes 2, 4, 13, 14, and 15.

### Genomic Predictions

**Figure 3:**
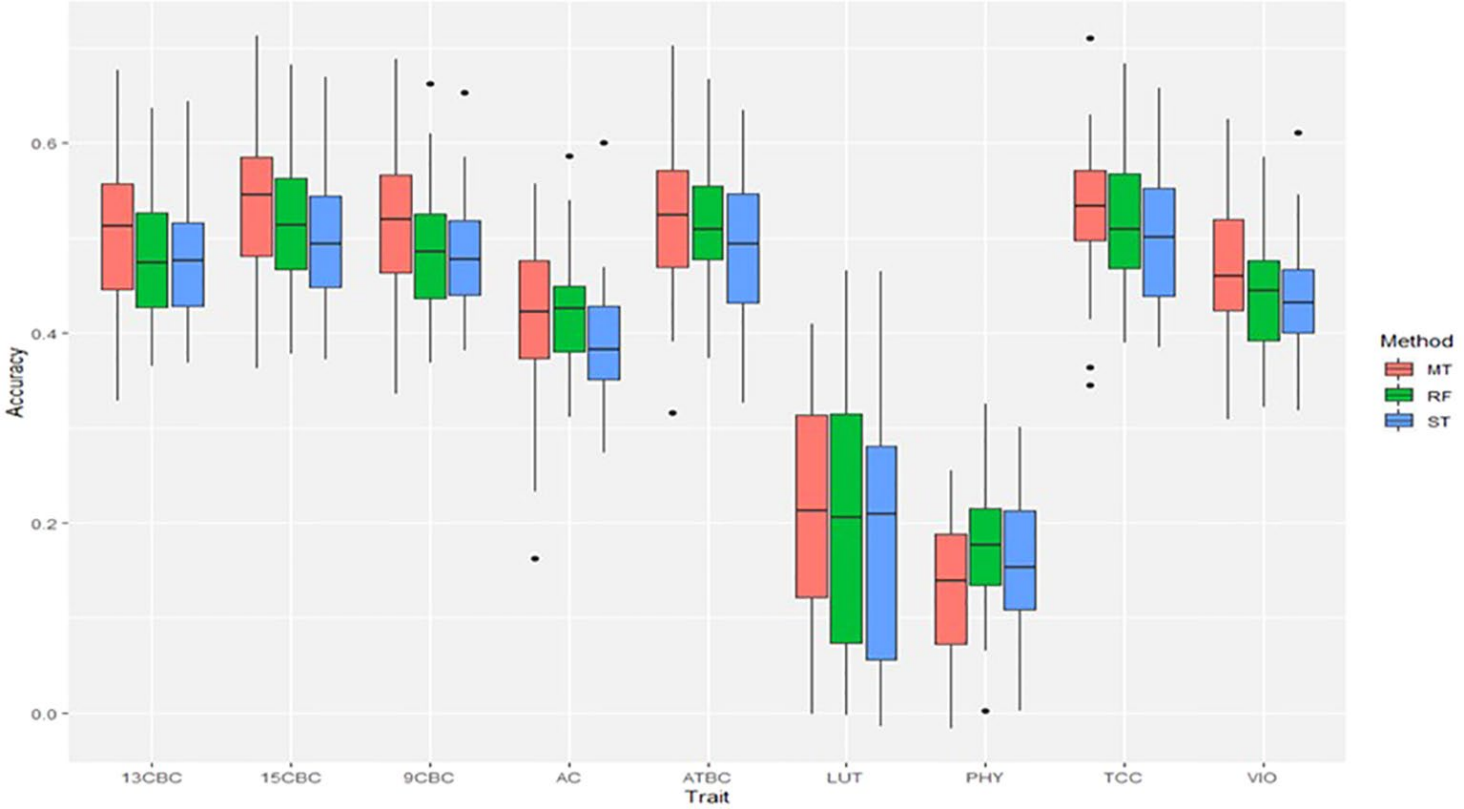
Genomic predictions for total carotenoid content (TCC), all-trans β-carotene (ATBC), violaxanthin (VIO), lutein (LUT), 15-cis beta-carotene (15CBC), 13-cis beta-carotene (13CBC), alpha-carotene (AC), 9-cis beta-carotene (9CBC), and phytoene (PHY) cassava root carotenoids. For each trait: ST = single trait GBLUP, RF = single trait random forest and MT = multiple-trait GBLUP models.

The result of the genomic predictions for the studied carotenoids showed a slight increase in prediction accuracies using ST-RF compared to the linear ST-GBLUP models (**Figure 3**). On the other hand, the MT-GBLUP models had slightly higher accuracies than the ST-RF model except in 9CBC and 13CBC where the accuracies were similar. Overall, prediction accuracies ranged from 0.16 in PHY to 0.52 in TCC (**Figure 3**).

## Discussion

Robust calibration performance has been previously reported using the same calibration set that was used in this study (Ikeogu et al., 2017). It is important to note that both the CIAT calibration set and the NRCRI test set were mixed, containing cassavas characterized as white and yellow. Trait heritabilities, correlations, and prediction accuracies reported here are therefore valid only for such mixed populations, which would not be typical of breeding populations. The use of ST-RF in this study was valuable in accounting for any potential nonlinear relationship between the variables (Svetnik et al., 2003; Lee et al., 2012; Ghasemi and Tavakoli, 2013). Most importantly, it was relevant in restricting negative prediction of constituents by using average prediction technique obtained from several trees of RF (Breiman et al., 1984; Qi, 2012). The coefficient of correlation (r) and determination (R^2^) have been used in assessing calibration performance (Duan et al., 2012; Wang et al., 2014) and the values obtained in this study (**Table 1**), which are similar to previous calibration results with linear models, are most valuable for screening and quantification of constituents (Cai et al., 2012; Fox et al., 2012; Lebot, 2012). Nevertheless, there is still a need for calibration improvement. Possible adoption of specific mathematical treatments for each trait, increasing the number of calibration samples and the use of variable selection approaches could potentially help to improve the current calibration models (Centner et al., 1996; Tosato et al., 2016). Also, the revalidation model approach and the use of local regression could be useful in improving predictions particularly when the target constituents evolve in breeding programs (Davrieux et al., 2016). This initial calibration and the application of NIRS in the assessment of traits in a low-resource national breeding program is promising especially when there are no cost-effective and efficient alternatives for such evaluations. This study provides an opportunity for rapid improvement of many valuable traits in cassava.

Several studies have shown that TCC is a highly heritable trait in cassava (Morillo et al., 2012; Ceballos et al., 2013; Esuma et al., 2016; Rabbi et al., 2017). Besides TCC, we observed moderately high heritability for most of the carotenoids, though it is unclear what heritabilities might be in a population composed only of yellow cassavas (Ceballos et al., 2013). High heritability for TCC and the individual carotenoid components has been reported in maize (Kandianis et al., 2013).

Understanding the genetic relationship especially between TCC and its components is vital in assessing the amount of progress made so far or required for a simultaneous increase TCC and its corresponding components, most especially, the PVAC in cassava. Previous efforts, especially in most of the low resource breeding programs, have centered mainly on the qualitative improvement of TCC partly due to the lack of effective and standardized phenotyping protocols. The high and positive phenotypic and genotypic relationships observed between TCC and the PVAC (especially AC, ATBC, VIO, 9CBC, 13CBC, and 15CBC) were encouraging and suggested that these traits could be improved concurrently. Because of its health benefits, the positive and significant genetic association between LUT and the PVAC (**Figure 1**) have a favorable implication for the health of millions of people that depend on cassava as a major staple. Lutein is a very important component of the macular pigment in the eyes and its deficiency is closely associated with some eye-related problems (Krinsky and Johnson, 2005; Kim et al., 2010; Bechoff et al., 2015). The positive associations offer the opportunity for simultaneous improvement of these traits and improving the nutritional value and health of millions of cassava users, especially women and children in sub-Saharan Africa. However, the low and significantly negative association between PHY and LUT requires further biochemical and genetic insights, including the design of adequate strategies in improving these traits. High and positive correlations especially between AC and PHY as well as between AC and ATBC have been reported in carrot (Santos et al., 2005). Although a negative interaction between β-carotene and lutein was reported, the positive interaction between β-carotene and concentrations of α-carotene were in agreement with previously reported bioavailability and bioconversion studies in carotenoids (van Vliet et al., 1996; Castenmiller and West, 1998).

The GWAS result was in agreement with the previous GWAS reports on TCC in cassava (Esuma et al., 2016; Rabbi et al., 2017). The identified candidate gene, phytoene synthase gene (*Manes.01G124200*) has been reported as a single genomic region associated with quantitative variation in TCC using both a panel of partial S1 and S2 inbreds (Esuma et al., 2016) and a diverse African germplasm collection phenotyped using an indirect color chart and a Chromameter value (Rabbi et al., 2017). However, other than the single major locus associated with qualitative or quantitative measures of TCC, the possibility of more than one associated locus has been widely suggested (Iglesias et al., 1997; Akinwale et al., 2010; Esuma et al., 2016; Rabbi et al., 2017). Previous genetic study of the progeny (F2 population) of a cross between yellow and white parents suggested that yellowness in cassava is controlled by two major genes, one controlling the transport of the product of precursors to the roots and the other responsible for the accumulation process (Chavez et al., 2000). This study uncovered additional regions for variation in TCC as well as the individual carotenoids. We identified regions that are significant for more than a single carotenoid which suggests the possibility of pleiotropic effects. Epistatic effects of the major genes had been earlier reported for TCC in cassava (Chavez et al., 2000). Some evidence of pleiotropic effects on multiple carotenoids have been reported by various genetic mapping studies especially in maize (Harjes et al., 2008; Yan et al., 2010; Kandianis et al., 2013). However, further investigations will be necessary to fully understand the physiological processes and interactions surrounding the carotenoid biosynthetic pathway in cassava (Mayfield et al., 1986; Shumskaya and Wurtzel, 2013).

The benefits of GS as a breeding tool in reducing breeding cycle time and accelerating the rate of genetic gain, especially that of complex traits, has been demonstrated in cassava (Oliveira et al., 2014; Okeke et al., 2017; Wolfe et al., 2017). GS has been widely used in many plants and animal breeding programs (Lorenz et al., 2011; Daetwyler et al., 2013; Zhang et al., 2015) and its adoption in cassava improvement is vital in fast-tracking product delivery in terms of varieties to meet the food and upcoming industrial demand for the crop. On average, we obtained higher prediction accuracies with the multiple-trait GBLUP while the nonlinear single trait RF had higher accuracies than the linear single trait GBLUP models. Multiple-trait models use the estimate of genetic and residual covariance in deriving GEBV for the traits of interest (Jia and Jannink, 2012; Okeke et al., 2017; Montesinos-Lopez et al., 2018). The benefit of multiple-trait models is very effective especially in the joint analyses of low and high heritable traits with medium to high genetic correlations (Calus and Veerkamp, 2011; Okeke et al., 2017). The advantage of nonlinear GS over linear models has been widely reported (Heslot et al., 2012; Pérez-Rodríguez et al., 2012; Crossa et al., 2014). Nonlinear models help to capture dominance and epistatic effects and enable the prediction of TGV rather than GEBV (Spindel et al., 2015; Wolfe et al., 2017). The prediction of TGV is valuable for crops like cassava and rice where released varieties are clones and inbreds, respectively.

Although genotyping costs are drastically decreasing, it could still be considered relatively expensive for resource-limited breeding programs to genotype a large collection of genetic materials, especially at the early breeding generations. Offsetting the high genotyping and classical phenotyping costs in such setups, NIRS provides an opportunity to incorporate certain descriptors in improving genomic predictions and overall breeding cost-efficiency (Hayes et al., 2017). Near-infrared spectroscopy wavelengths significant for some important traits could be targeted for candidate genes. The use of NIRS as a high-throughput, low cost, and nondestructive tool in the indirect capture of endophenotypic variants and the computation of relationship matrices for predicting complex traits has been suggested (Rincent et al., 2018) and this will be a very useful concept for low-resource breeding programs. The combination of rapid phenotyping using NIRS and the adoption of genomic breeding tools in cassava will lead to the reduction of phenotyping cost and time, enable the addition of more individuals for selection, promote genetic diversity, and shorten breeding cycle time. Due to its flexibility, NIRS can be useful in tracking carotenoid concentrations in cassava roots before and after processing. This is important in the current effort in increasing the content of carotenoids in a crop where increases in fresh weight gains need to be translated into dry weight in the final cassava products, given that the relationship between carotenoid concentrations on fresh and dry weight basis is not always linear (Iglesias et al., 1997; Ceballos et al., 2017).

## Conclusion

This study complements the current effort in addressing vitamin A deficiency in many regions of the world through the bio-fortification of major staple foods (Chávez et al., 2005; Pillay et al., 2014). The quantitative evaluation of total and individual carotenoids offers a tremendous opportunity in understanding the natural genetic diversity and the underlying architecture of these traits in cassava. The positive and high genotypic associations observed in this study underscores the fact that any effort in increasing TCC could lead to an increase in the individual components. Such information is beneficial in designing the best strategy for improving carotenoids content in cassava (Bouis et al., 2011; Ceballos et al., 2013). The identified loci associated with variation in carotenoids could be used in MAS for improved nutritional quality in cassava. Also, the information from the GWAS analysis could be incorporated into GS to improve predictions of carotenoid content in the genetic background of other relevant agronomic traits (Spindel et al., 2015; Wolfe et al., 2016).

This study supports the usefulness of GS in accelerating the improvement of carotenoids in cassava as demonstrated in other traits and species (Hayes et al., 2010; Ly et al., 2013; Hayes et al., 2017; Wolfe et al., 2017). The use of nonlinear GS models has the potential to capture nonlinear underlying relationships between dependent and independent variables and are beneficial in predicting TGVs in cassava (Heslot et al., 2012; Wolfe et al., 2017). In addition, the use of multiple-trait models could help improve GS prediction accuracies.

## Data Availability Statement

The datasets generated for this study can be found in the ftp://ftp.cassavabase.org/manuscripts/Ikeogu_et_al_2019. 

## Author Contributions

As part of UI PhD proposal, CE, UO, and J-LJ contributed in the initial discussion and design of the study; UI and AC were involved in data collection while UI, DA, MW, and UO contributed in statistical analyses; UI wrote the first draft and while all the other authors participated in manuscript revision; J-LJ, DA, and CE approved the final submission as revised by UI.

## Funding

Funding for this work was provided by the Bill and Melinda Gates Foundation and UKAID (Grant 1048542, http://www.gatesfoundation.org) as part of the Next Generation Cassava Breeding project and the PhD degree of Ugochukwu N Ikeogu.

## Conflict of Interest

The authors declare that the research was conducted in the absence of any commercial or financial relationships that could be construed as a potential conflict of interest.
