# Supplementary material for: Genetic Correlation, Genome-Wide Association and Genomic Prediction of Portable NIRS Predicted Carotenoids in Cassava Roots
Source: Front Plant Sci. 2019 Dec 4;10:1570. doi: 10.3389/fpls.2019.01570 (PMC6904298; doi:10.3389/fpls.2019.01570)
Supplement: Supplementary file 1 [file Table_1.docx]

**Table S1: Markers with genome-wide association significance for carotenoids in cassava roots.**

| Trait | Marker | Chr | Pos. (Mb) | Allele | Freq. | SNP Effect | p-value |
| --- | --- | --- | --- | --- | --- | --- | --- |
| ATBC | 1_23386060 | 1 | 23.39 | T/C | 0.28 | 0.385 | 4.94E-07 |
| ATBC | 1_24105199 | 1 | 24.11 | C/A | 0.32 | 0.438 | 3.69E-08 |
| ATBC | 1_24181156 | 1 | 24.18 | A/G | 0.47 | 0.328 | 5.51E-08 |
| ATBC | 1_24611696 | 1 | 24.61 | T/G | 0.31 | 0.432 | 4.36E-08 |
| ATBC | 1_24636113 | 1 | 24.64 | G/A | 0.31 | 0.400 | 4.36E-07 |
| ATBC | 1_24709749 | 1 | 24.71 | T/A | 0.31 | 0.416 | 1.23E-07 |
| ATBC | 13_16164208 | 13 | 16.16 | T/A | 0.05 | 0.592 | 8.98E-08 |
| LUT | 1_4814833 | 1 | 4.81 | G/T | 0.05 | 0.031 | 1.98E-07 |
| LUT | 1_4815271 | 1 | 4.82 | T/A | 0.06 | 0.027 | 1.42E-07 |
| LUT | 1_4857197 | 1 | 4.86 | C/A | 0.06 | 0.032 | 1.13E-08 |
| LUT | 1_17482176 | 1 | 17.48 | A/G | 0.06 | 0.032 | 8.78E-09 |
| LUT | 4_22536069 | 4 | 22.54 | T/G | 0.06 | 0.030 | 2.62E-07 |
| LUT | 4_22974535 | 4 | 22.97 | G/A | 0.06 | 0.030 | 1.25E-07 |
| LUT | 4_23362863 | 4 | 23.36 | A/G | 0.05 | 0.034 | 3.96E-08 |
| LUT | 4_23367235 | 4 | 23.37 | C/T | 0.06 | 0.030 | 2.01E-07 |
| LUT | 4_23693408 | 4 | 23.69 | G/A | 0.05 | 0.034 | 2.78E-08 |
| LUT | 13_6088827 | 13 | 6.09 | T/A | 0.09 | 0.025 | 1.04E-07 |
| LUT | 14_24239911 | 14 | 24.24 | T/C | 0.05 | 0.031 | 3.28E-07 |
| LUT | 15_14171698 | 15 | 14.17 | G/A | 0.05 | 0.031 | 1.86E-08 |
| TCC | 1_23386060 | 1 | 23.39 | T/C | 0.28 | 0.512 | 1.77E-07 |
| TCC | 1_24105199 | 1 | 24.11 | C/A | 0.32 | 0.606 | 2.88E-09 |
| TCC | 1_24117585 | 1 | 24.12 | C/G | 0.35 | 0.488 | 4.77E-08 |
| TCC | 1_24121247 | 1 | 24.12 | T/C | 0.36 | 0.464 | 8.54E-08 |
| TCC | 1_24139256 | 1 | 24.14 | C/T | 0.48 | 0.449 | 2.71E-07 |
| TCC | 1_24140688 | 1 | 24.14 | G/C | 0.48 | 0.473 | 7.08E-08 |
| TCC | 1_24159583 | 1 | 24.16 | T/C | 0.36 | 0.443 | 5.05E-07 |
| TCC | 1_24181156 | 1 | 24.18 | A/G | 0.47 | 0.442 | 1.04E-08 |
| TCC | 1_24238287 | 1 | 24.24 | T/C | 0.45 | 0.450 | 3.92E-07 |
| TCC | 1_24239005 | 1 | 24.24 | G/A | 0.44 | 0.476 | 1.58E-07 |
| TCC | 1_24272965 | 1 | 24.27 | G/C | 0.41 | 0.465 | 5.51E-07 |
| TCC | 1_24315496 | 1 | 24.32 | G/A | 0.40 | 0.443 | 1.97E-07 |
| TCC | 1_24611696 | 1 | 24.61 | T/G | 0.31 | 0.597 | 3.80E-09 |
| TCC | 1_24614646 | 1 | 24.61 | C/A | 0.35 | 0.435 | 2.57E-07 |
| TCC | 1_24636113 | 1 | 24.64 | G/A | 0.31 | 0.572 | 1.80E-08 |
| TCC | 1_24653227 | 1 | 24.65 | C/G | 0.32 | 0.570 | 1.14E-08 |
| TCC | 1_24709749 | 1 | 24.71 | T/A | 0.31 | 0.572 | 1.46E-08 |
| TCC | 2_12738969 | 2 | 12.74 | A/G | 0.06 | 0.749 | 4.71E-07 |
| TCC | 13_2185403 | 13 | 2.19 | G/C | 0.15 | 0.591 | 4.34E-07 |
| TCC | 13_2185406 | 13 | 2.19 | A/T | 0.15 | 0.591 | 4.34E-07 |
| VIO | 1_25426915 | 1 | 25.43 | T/G | 0.36 | 0.010 | 3.50E-07 |
| 13CBC | 1_24105199 | 1 | 24.11 | C/A | 0.32 | 0.053 | 3.37E-08 |
| 13CBC | 1_24117585 | 1 | 24.12 | C/G | 0.35 | 0.047 | 2.41E-08 |

**Table S1 (Continued)**

| 13CBC | 1_24121247 | 1 | 24.12 | T/C | 0.36 | 0.048 | 5.81E-09 |
| --- | --- | --- | --- | --- | --- | --- | --- |
| 13CBC | 1_24121295 | 1 | 24.12 | T/C | 0.36 | 0.043 | 1.92E-07 |
| 13CBC | 1_24121316 | 1 | 24.12 | T/A | 0.36 | 0.043 | 1.81E-07 |
| 13CBC | 1_24139256 | 1 | 24.14 | C/T | 0.48 | 0.045 | 6.23E-08 |
| 13CBC | 1_24140688 | 1 | 24.14 | G/C | 0.48 | 0.044 | 1.01E-07 |
| 13CBC | 1_24159583 | 1 | 24.16 | T/C | 0.36 | 0.046 | 4.51E-08 |
| 13CBC | 1_24160008 | 1 | 24.16 | G/A | 0.35 | 0.043 | 2.88E-07 |
| 13CBC | 1_24181156 | 1 | 24.18 | A/G | 0.47 | 0.040 | 3.85E-08 |
| 13CBC | 1_24239005 | 1 | 24.24 | G/A | 0.44 | 0.043 | 5.52E-07 |
| 13CBC | 1_24611696 | 1 | 24.61 | T/G | 0.31 | 0.055 | 1.01E-08 |
| 13CBC | 1_24614646 | 1 | 24.61 | C/A | 0.35 | 0.045 | 2.30E-08 |
| 13CBC | 1_24632970 | 1 | 24.63 | T/C | 0.37 | 0.039 | 5.29E-07 |
| 13CBC | 1_24636113 | 1 | 24.64 | G/A | 0.31 | 0.053 | 3.40E-08 |
| 13CBC | 1_24653227 | 1 | 24.65 | C/G | 0.32 | 0.057 | 1.44E-09 |
| 13CBC | 1_24663824 | 1 | 24.66 | G/C | 0.35 | 0.042 | 1.78E-07 |
| 13CBC | 1_24664143 | 1 | 24.66 | A/G | 0.35 | 0.045 | 4.89E-08 |
| 13CBC | 1_24709749 | 1 | 24.71 | T/A | 0.31 | 0.053 | 2.97E-08 |
| 13CBC | 1_25426915 | 1 | 25.43 | T/G | 0.36 | 0.040 | 5.71E-07 |
| 13CBC | 13_2185403 | 13 | 2.19 | G/C | 0.15 | 0.056 | 4.47E-07 |
| 13CBC | 13_2185406 | 13 | 2.19 | A/T | 0.15 | 0.056 | 4.47E-07 |

**Table S1 (Continued)**

| 13CBC | 13_2208957 | 13 | 2.21 | A/G | 0.14 | 0.058 | 5.23E-07 |
| --- | --- | --- | --- | --- | --- | --- | --- |
| 15CBC | 1_23386060 | 1 | 23.39 | T/C | 0.28 | 0.010 | 1.44E-07 |
| 15CBC | 1_24105199 | 1 | 24.11 | C/A | 0.32 | 0.011 | 3.37E-09 |
| 15CBC | 1_24117585 | 1 | 24.12 | C/G | 0.35 | 0.009 | 2.00E-08 |
| 15CBC | 1_24121247 | 1 | 24.12 | T/C | 0.36 | 0.009 | 1.64E-08 |
| 15CBC | 1_24121295 | 1 | 24.12 | T/C | 0.36 | 0.008 | 2.56E-07 |
| 15CBC | 1_24121316 | 1 | 24.12 | T/A | 0.36 | 0.008 | 2.77E-07 |
| 15CBC | 1_24139256 | 1 | 24.14 | C/T | 0.48 | 0.009 | 4.31E-08 |
| 15CBC | 1_24140688 | 1 | 24.14 | G/C | 0.48 | 0.009 | 2.84E-08 |
| 15CBC | 1_24159583 | 1 | 24.16 | T/C | 0.36 | 0.009 | 8.40E-08 |
| 15CBC | 1_24160008 | 1 | 24.16 | G/A | 0.35 | 0.008 | 4.47E-07 |
| 15CBC | 1_24181156 | 1 | 24.18 | A/G | 0.47 | 0.008 | 5.99E-09 |
| 15CBC | 1_24224353 | 1 | 24.22 | T/G | 0.48 | 0.008 | 5.10E-07 |
| 15CBC | 1_24239005 | 1 | 24.24 | G/A | 0.44 | 0.009 | 1.70E-07 |
| 15CBC | 1_24611696 | 1 | 24.61 | T/G | 0.31 | 0.011 | 4.84E-09 |
| 15CBC | 1_24614646 | 1 | 24.61 | C/A | 0.35 | 0.008 | 5.64E-08 |
| 15CBC | 1_24636113 | 1 | 24.64 | G/A | 0.31 | 0.010 | 2.44E-08 |
| 15CBC | 1_24653227 | 1 | 24.65 | C/G | 0.32 | 0.011 | 3.76E-09 |
| 15CBC | 1_24664143 | 1 | 24.66 | A/G | 0.35 | 0.008 | 1.65E-07 |
| 15CBC | 1_24709749 | 1 | 24.71 | T/A | 0.31 | 0.011 | 1.09E-08 |
| 15CBC | 13_2185403 | 13 | 2.19 | G/C | 0.15 | 0.011 | 1.45E-07 |

**Table S1 (Continued)**

| 15CBC | 13_2185406 | 13 | 2.19 | A/T | 0.15 | 0.011 | 1.45E-07 |
| --- | --- | --- | --- | --- | --- | --- | --- |
| 9CBC | 1_24105199 | 1 | 24.11 | C/A | 0.32 | 0.043 | 3.50E-08 |
| 9CBC | 1_24117585 | 1 | 24.12 | C/G | 0.35 | 0.038 | 3.32E-08 |
| 9CBC | 1_24121247 | 1 | 24.12 | T/C | 0.36 | 0.038 | 9.33E-09 |
| 9CBC | 1_24121295 | 1 | 24.12 | T/C | 0.36 | 0.034 | 3.38E-07 |
| 9CBC | 1_24121316 | 1 | 24.12 | T/A | 0.36 | 0.034 | 2.88E-07 |
| 9CBC | 1_24139256 | 1 | 24.14 | C/T | 0.48 | 0.036 | 3.83E-08 |
| 9CBC | 1_24140688 | 1 | 24.14 | G/C | 0.48 | 0.036 | 6.47E-08 |
| 9CBC | 1_24159583 | 1 | 24.16 | T/C | 0.36 | 0.036 | 1.05E-07 |
| 9CBC | 1_24181156 | 1 | 24.18 | A/G | 0.47 | 0.032 | 3.47E-08 |
| 9CBC | 1_24611696 | 1 | 24.61 | T/G | 0.31 | 0.044 | 8.18E-09 |
| 9CBC | 1_24614646 | 1 | 24.61 | C/A | 0.35 | 0.036 | 2.76E-08 |
| 9CBC | 1_24636113 | 1 | 24.64 | G/A | 0.31 | 0.042 | 4.96E-08 |
| 9CBC | 1_24653227 | 1 | 24.65 | C/G | 0.32 | 0.045 | 3.01E-09 |
| 9CBC | 1_24663824 | 1 | 24.66 | G/C | 0.35 | 0.034 | 1.93E-07 |
| 9CBC | 1_24664143 | 1 | 24.66 | A/G | 0.35 | 0.036 | 4.86E-08 |
| 9CBC | 1_24709749 | 1 | 24.71 | T/A | 0.31 | 0.042 | 3.14E-08 |
| 9CBC | 13_2185403 | 13 | 2.19 | G/C | 0.15 | 0.045 | 5.04E-07 |
| 9CBC | 13_2185406 | 13 | 2.19 | A/T | 0.15 | 0.045 | 5.04E-07 |
